# Optoacoustic lenses for lateral sub-optical resolution elasticity imaging

**DOI:** 10.1016/j.pacs.2024.100663

**Published:** 2024-11-16

**Authors:** Mengting Yao, Rafael Fuentes-Domínguez, Salvatore La Cavera, Fernando Pérez-Cota, Richard J. Smith, Matt Clark

**Affiliations:** Optics and Photonics Group, Faculty of Engineering, University of Nottingham, University Park, Nottingham, NG7 2RD, Nottinghamshire, United Kingdom

**Keywords:** 78A60, 92C55, Picosecond laser ultrasonics, Brillouin scattering, Phonon focusing, Elasticity imaging

## Abstract

In this paper, we demonstrate for the first time the focusing of gigahertz coherent phonon pulses propagating in water using picosecond ultrasonics and Brillouin light scattering. We achieve this by using planar Fresnel zone plate and concave lenses with different focal lengths. Pump light illuminating the optoacoustic lens generates a focusing acoustic field, and Brillouin scattered probe light allows the acoustic field to be continuously monitored over time. Agreement of the experiment with a numerical model suggests that we can generate a focused acoustic beam down to ∼250 nm. A clear focusing effect is observed experimentally as a modulation of the envelope of the time-resolved Brillouin scattering (TRBS) signal. These findings are a crucial step toward their application in high-resolution acoustic microscopy. This work experimentally demonstrates a method to narrow the lateral size of picosecond laser-generated phonon fields in an aqueous environment, making it well-suited for 3D imaging applications in biological systems using TRBS.

## Highlights

• We present optoacoustic lenses with the potential for super-optical resolution elasticity microscopy.
• Micro lens designs to produce acoustic focus with lateral dimensions down to ∼250 nm.
• First experimental demonstration of Brillouin scattering from focused phonons in water.
• Compatible with fibre probe endoscopy for enhanced versatility.

## Graphical abstract

**Figure:**
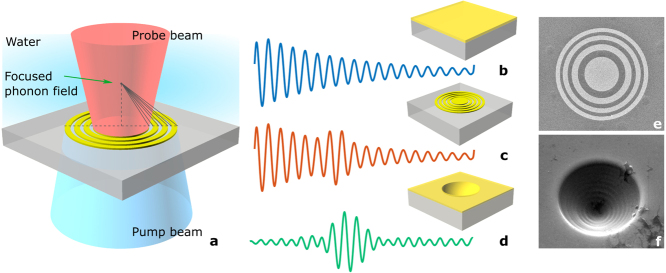

## 1 Introduction

Despite a long history of applications for the imaging and detection of macroscopic objects, acoustics holds great promise for high resolution imaging on the microscopic or even nanoscopic scale. Sound waves carry significantly less energy than light waves in most applications, from metals to soft biological tissue. Although both photons and phonons can accumulate energy in large quantities, a single optical photon carries five orders of magnitude more energy than an acoustic phonon. This becomes particularly relevant when short optical wavelengths are needed to realise high resolution imaging. In optics this issue leads to the well-known effects of biological phototoxicity, particularly for wavelengths in the near ultraviolet (NUV) or shorter portion of the electromagnetic spectrum, and has motivated a wave of fluorescent label-based super-resolution imaging techniques [1], [2]. However, in acoustics, generating and detecting acoustic waves with equivalent wavelengths (and therefore resolution) to NUV photons has met significant technical challenges [3]. Nonetheless a range of piezoelectric [4], [5], [6] and photoacoustic-based [7] techniques have been developed over the years which readily demonstrate the effects of acoustic focusing, albeit on the scales of MHz frequencies, which limits spatial resolution to the order of ∼1 mm.

Scanning acoustic microscopy (SAM) – which made use of concave acoustic lenses and high frequency piezoelectric transducers – offered a path for acoustics towards high resolution imaging and demonstrated proof of concept on a range of biological cells [8], [9], [10]. However, this collection of techniques suffered from a number of challenges. Firstly, acoustic wavelengths can be scaled to ∼100 nm by utilising higher frequency piezoelectric transducers (e.g., in the GHz range); although wavelength and resolution scale inversely with frequency (f), the attenuation of the waves in aqueous tissue scales with $f^{2}$ leading to prohibitive losses and short acoustic penetration depth. To circumvent this, lower frequency transducers (e.g., ∼100 MHz) can be used for greater penetration depth. Alternatively, to maintain high frequency, the sample temperature can be lowered, such as by subjecting the specimen to cryogenic conditions, although this limits the technique’s applicability to living biological samples.

Recent advances in optically-driven acoustic techniques, such as laser ultrasound [11], [12] and photoacoustics [13], [14], [15], [16], have offered solutions to some of the previous challenges of SAM. The photoacoustic generation of acoustic waves relies on the intrinsic absorption of pump light, therefore greatly simplifying transducer design and fabrication. Photoacoustic transmitters do not require electrical connectivity and are more robust to excitation than their fragile piezoelectric counterparts [17]. These improvements have reignited interest in achieving sub-micron wavelength acoustics. For instance, Che et al. developed an optically excited and detected concave acoustic lens which accessed sub-micron features of a grating [18] in a manner similar to SAM. However, this technique suffered from mechanical instabilities, especially in the stage, as well as a low signal-to-noise ratio. All pulse-echo-based techniques require that the acoustic field propagates both to and from the imaging object, a problem which is exacerbated at GHz frequencies, and consequentially lowers the achievable signal-to-noise ratio of the measurement.

Time-resolved Brillouin scattering (TRBS), also referred to time-domain Brillouin scattering (TDBS) and picosecond acoustic interferometry [19], [20], [21], [22], [23], beats the attenuation of pulse-echo techniques by at least factor of two because the acoustic field is measured directly in the sample volume. In TRBS the propagating acoustic field photoelastically induces a change in refractive index that optically scatters a probe laser beam. This makes possible non-contact detection of GHz frequency phonons without the need for long acoustic path length pulse-echo configurations, therefore partially mitigating the effects of acoustic attenuation. TRBS has been used in a microscopy capacity to demonstrate high contrast high resolution label-free mechanical imaging over a range of biological and solid-state samples [24], [25], [26], [27], [28], [29], [30]. Dehoux et al. utilised TRBS to detect the focusing of GHz acoustic fields within the cylindrical cross-section of silica optical fibres [31]. However, this important proof of concept is not readily translatable to conventional microscopy environments containing liquid medium.

Here we present a novel approach to super-optical resolution acoustic microscopy based on picosecond ultrasonics and Brillouin scattering. We propose and demonstrate micro-lens designs that enable us to experimentally observe the focusing of coherent acoustic phonons in aqueous media in normal environmental conditions. These lenses, including flat Fresnel zone plates and concave structures, pave the way for TRBS-based super-optical elasticity imaging applications.

## 2 Methods

### 2.1 Working principle

**Fig. 1:**
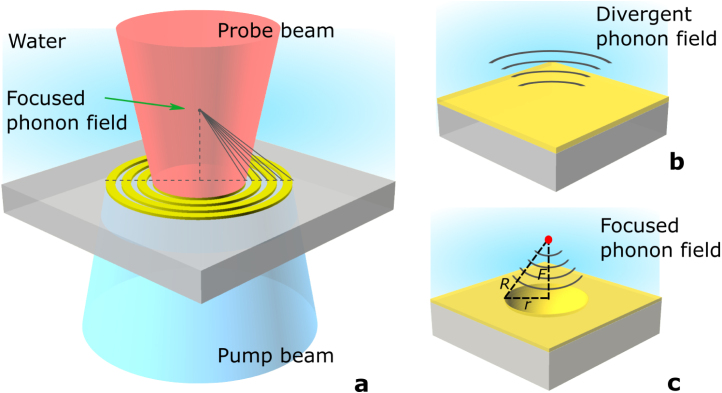
Schematic illustration of the experimental setup and different transducer configurations. (a) The experimental setup for generating and probing a focused phonon field within a water medium using a Fresnel zone plate. The thin Fresnel zone plate, 20 nm gold on a glass substrate, absorbs the pump (blue) energy and generates a focused phonon field. A probe beam (red) detects the acoustic field. The pump beam is delivered from the bottom, and the probe beam is delivered from the top and measured in reflection. (b) Conventional flat transducer configuration, where the generated phonon field spreads outwards. (c) Concave lens configuration, showing the acoustic waves converging to a focal point, enhancing the localised intensity of the acoustic field.

Our approach integrates TRBS with lensing elements aiming to achieve super-optical resolution while being compatible with existing approaches used for live cell imaging (e.g., phonon microscopy [26], [32]) and simple enough to be fabricated on optical fibres (e.g., phonon probe endoscopy [29], [33]). Coherent acoustic phonons (CAPs) are generated using an ultrafast pulsed pump laser and optoacoustic lenses via a photoacoustic process. The propagation of these CAPs is detected via Brillouin scattering using another ultrafast pulsed probe laser, as illustrated in Fig. 1(a). Brillouin scattering provides a direct measurement of sound velocity, facilitating the quantification of elasticity through the measurement of Brillouin frequency [21], [34]. The Brillouin frequency $f_{B}$ is given by:

(1) $$f_{B}=\frac{2\nu n}{\lambda_{probe}}\cos\left(\theta \right),$$

where n is the refractive index, ν is the sound velocity, θ is the incident angle, and $\lambda_{probe}$ is the optical probing wavelength. By assuming a constant sound velocity in the homogeneous medium, the spatial location of the CAP in depth z can be obtained through the time of flight t using the relationship z=νt.

Two types of optoacoustic lenses, Fresnel Zone Plate (FZP) lenses and concave lenses are employed to generate converging sound, allowing for focusing to a volume smaller than that achievable with our specific microscope setup. FZP lenses use diffraction to create focus by replacing flat transducers (Fig. 1(b)) with annular rings (Fig. 1(a)). In contrast, concave lenses (Fig. 1(c)) achieve focus by shaping flat transducers with a concave curvature.

### 2.2 Design and fabrication of lenses

We have implemented two lens designs based on concave mirrors and Fresnel zone plates. Fresnel zone plates utilise zones of constructive and destructive interference to focus waves without the need for traditional lens materials or 3D structures as shown in Fig. 1(a). The radius and width of each ring can be calculated by [35], [36]:

(2) $$r_{k}=\sqrt{k\lambda_{a}F+\frac{{\left(k\lambda_{a}\right)}^{2}}{4}},\;k=1,2...N,$$

where F is the designed focal length and N is the total number of zones. The number of zones is directly linked to the acoustic wavelength and the diameter of the lens, with the total number of zones determined by how many rings fit within the lens diameter (set to ∼5 μm, matching the core size of a single-mode fibre). The width of each ring is inversely related to the zone number, decreasing as the zones move outward, ensuring phase alignment for effective focusing. The wavelength of the detected CAP $\lambda_{a}$ is determined by the wavelength of the probe laser $\lambda_{probe}$, according to the Bragg condition:

(3) $$\lambda_{a}=\frac{\lambda_{probe}}{2n}$$

Using Eqs. (2), (3) with $\lambda_{probe}=780\;\mathrm{nm}$ and n=1.33 ($\lambda_{a}=293\;\mathrm{nm}$) we designed an FZP with 8 zones (4 elements) and a range of focal lengths (F) from 1 to 4 μm. One design constraint is the smallest feature size that can be made. For instance, an FZP with F=2 μm requires that the smallest ring has a width of 190 nm.

To accommodate the small feature sizes of the FZP rings, electron beam lithography (EBL) was used for fabrication. An electron beam resist layer (AR-P 6200) and a safeguarding resist layer (AR-PC 5090) were applied to a glass coverslip. Subsequently, a soft baking procedure was carried out, followed by electron beam exposure using a Nanobeam nB5 electron beam lithography system with bespoke designs containing the pattern geometry calculated as described above. It was essential to add an extra protective and conductive resist layer to dissipate charges during the electron exposure to ensure the features remained sharp. Following this, the conductive protective layer was removed using deionised water. An adhesion layer of 5 nm of indium tin oxide (ITO) and a 20 nm layer of gold (Au) was applied using a thermal evaporator. The gold layer acts as the optoacoustic transduction layer. Finally, the lift-off procedure was completed using AR 600-546 to reveal the final patterns.

The layer thickness was selected to be between 20 and 30 nm (due to fabrication tolerances) to enable red probe light to pass through but be sufficiently absorbing for blue pump light to generate phonons.

We then explored a more conventional strategy for focusing acoustic waves using a concave transducer geometry. Assuming that the CAPs are emitted along surface normals to each position of the curved transducer surface, we can relate the design focal length to the lens radius. The combination of the radius of curvature and the lens aperture width will determine the effective numerical aperture of the acoustic lens. For example, in order to produce a lens with a focal length of 3 μm (from the flat surface of the substrate) and an aperture radius of 2.5 μm a surface corresponding to the surface of a sphere radius of 3.9 μm should be machined into the substrate. The radius of the sphere is given by $R=\sqrt{F^{2}+r^{2}}$ where F is the required focal length and r is the aperture radius (see Fig. 1(c)).

The 3D shape with variations in height needs the use of specialised fabrication techniques such as focused ion beam (FIB) milling, which allows for precise sculpting of the lens profile directly into the substrate material. Here a clean glass substrate was coated with a 30 nm layer of ITO as it is conductive and reduced the effect of charge build up during the milling process which would otherwise unduly affect the resolution of the patterning. An FEI Quanta 200 3D Dual Beam FIB machine, with a 10 nm (30 kV) at 1 pA ion beam resolution, was then used to create the dish by removing circles of decreasing radius from the surface of the sample [37]. Each dish was typically divided into 15 layers. After milling, an adhesion layer of ITO was added to the sample, and then the whole sample was over-coated with 30 nm of gold to act as the optoacoustic transduction layer.

### 2.3 Simulations

We have developed a simple model that uses the Fourier–Bessel angular spectrum propagation (FBASP) to calculate field propagation for both light and sound [38], [39]. This model has the advantage of being less computationally demanding. The propagation of both waves is simplified by assuming radial symmetry and a single acoustic frequency (optical probing wavelength $\lambda_{probe}$ defines the sound frequency $f_{B}$ as in Eq. (1), by assuming plane wave for both the acoustic and probe fields interacting at a single angle θ). In reality, the physical CAP generation process is broadband, however, the Brillouin scattering interaction acts as an effective bandpass filter centred on the Bragg wavelength (see Eq. (3)). The model first defines the NA of both the pump and probe objective lenses. Then the point spread functions (PSF) for both beams are calculated using an Airy disk assumption ($\lambda_{pump}=415$ nm, NA=0.1, $d_{pump}=5.1$ μm and $\lambda_{probe}=780$ nm, NA = 0.3, $d_{probe}=$ 2.3 μm, beam radius find in intensity at the airy disk assumption).

The amplitude of the initial sound field transmitted into the medium is proportional to the pump PSF for a conventional flat transducer. However, when a lens structure is introduced (FZP or concave structures), in addition to the amplitude distribution, the acoustic phase at the initial plane $z_{0}$ is also modified using the phase change from the geometry of the lenses. From this, we get the initial sound and probe fields $u_{sound}\left(r,z_{0}\right)$ and $u_{probe}\left(r,z_{0}\right)$, where $z_{0}$ is the Au–water interface chosen to be the phase origin of acoustic and optical fields and r is the radius. Then the optical and acoustic fields are propagated along the optical axis z to estimate field amplitude and phase using FBASP [38], [39].

**Fig. 2:**
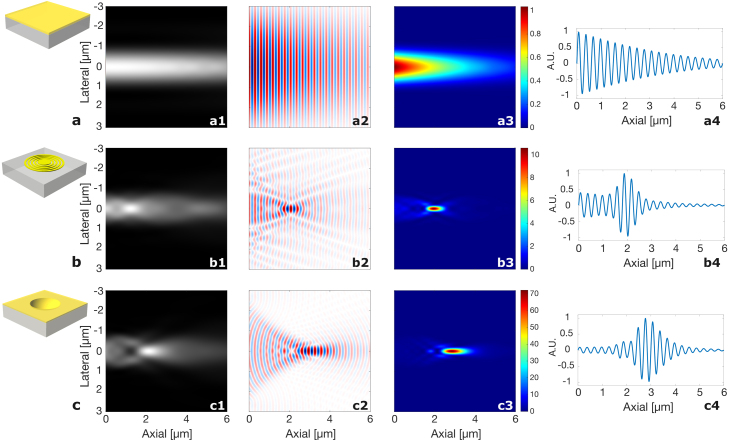
Simulated probe intensity, acoustic field, and overlapping field of the optical and acoustic fields for different transducer configurations. First row: (a) a conventional flat transducer. Second row: (b) a Fresnel zone plate structure with F = 2 μm. Third row: (c) a dished structure with F = 3 μm. First column: (1) simulated optical probe intensity computed using FBASP. Second column: (2) simulated acoustic field amplitude computed using FBASP. Third column: (3) Overlap of the optical probe and sound fields (optical amplitude×acoustic amplitude [39], [40]). Fourth column: (4) simulated TRBS signal received by a large photodiode integrating all the received light. It can be seen that both the Fresnel zone plate and concave lenses show significant localisation of the amplitude of the TRBS signal around the focal point. In all cases the radius of the pump beam was 2.5 μm.

The rows in Fig. 2 show the simulation results of the three transducer configurations: (a) flat transducer, (b) Fresnel zone plate, and (c) concave lens. For each configuration, the columns show (1) probe intensity, (2) acoustic field, (3) optical and acoustic overlap map — multiplication of the optical intensity and acoustic amplitude [40], and (4) expected TRBS trace (the integral along r of the map in column (3)). The flat transducer reflects the incoming probe (a1), and the pump-generated acoustic field is non-focusing (a2). The overlap map (a3) and the simulated TRBS signal (a4) exhibit an exponential decay. The Fresnel zone plate lens reflects a portion of the incoming probe beam (b1) and focuses the CAP at the designed focal point (b2), 2 μm. The overlap map indicates a tight focus (b3), and the simulated TRBS trace shows an increased signal envelope (b4). The curvature of the concave lens creates a focused acoustic field (c2) while also reflecting and focusing the incoming probe light. Overlapping the acoustic and optical foci along the z-axis is important for obtaining an optimal signal. In the experiment, the optical focus was adjusted to achieve the best signal. To simulate this, the optical probe focus position, resulting from the reflection of the probe light off the acoustic lens, was adjusted axially to coincide with the acoustic focus. With both probe and sound focus overlapped, the overlap map and simulated TRBS trace exhibit the highest focusing efficiency at the designed focus 3 μm for this concave lens (c3, c4). Note that the FBASP model modifies the phase at the initial plane, $z_{0}=0$ μm, simulating only the propagation after the highest point of the structure, which corresponds to the unmilled flat substrate height of the concave lenses. However, in the following experimental measurements, TRBS optical tracking of CAP begins at the lens surface. As a result, the focus appears at an axial depth equal to the radius of the concave lens, for the F=3 μm lens, the radius is 3.9 μm. The simulation indicates that, without considering the detection objective’s NA, a focused acoustic beam waist of ∼224 nm and ∼280 nm can be achieved for the FZP and concave lens, respectively.

### 2.4 Experimental methods

The instrument is built around an asynchronous optical sampling system (ASOPS) with two short femtosecond laser pulses (∼100 fs, 80 MHz repetition rate). The pump (415 nm) is delivered and focused at the bottom of the transducers using a 5x objective lens (0.1 NA) which produces a ∼5 μm spot-size. The probe (780 nm) is delivered and focused at the top of the transducers using a 10x water dipping objective lens (0.3 NA) which produced a ∼2 μm spot-size. The reflected probe laser beam is collected using a photodiode through the same top objective that delivers the probe, using a quarter-wave plate, a beam splitter, and a half-wave plate for delivery and detection simultaneously, similar to the setup used in [39]. For FZP lenses, the measured TRBS signal is maximised using the flat Au transducer positioned close to the lens (corresponding to the large Au area in the EBL design). For concave lenses, the signal is optimised by adjusting the focus of the probe beam to achieve maximum overlap of the optical and acoustic fields in the centre of the lens.

The pump pulse is absorbed by a transducer layer (thin Au film); the absorbed light causes rapid heating and via thermal expansion launches CAPs into the sample. The TRBS signal arises due to the interference of the reflected unscattered probe laser beam with a portion scattered from the acoustic wave packet propagating in the sample. As the acoustic wave propagates, the phase of the scattered component changes relative to the non-scattered light producing an oscillating signal and allowing the detection of the propagating CAPs.

### 2.5 Signal processing

**Fig. 3:**
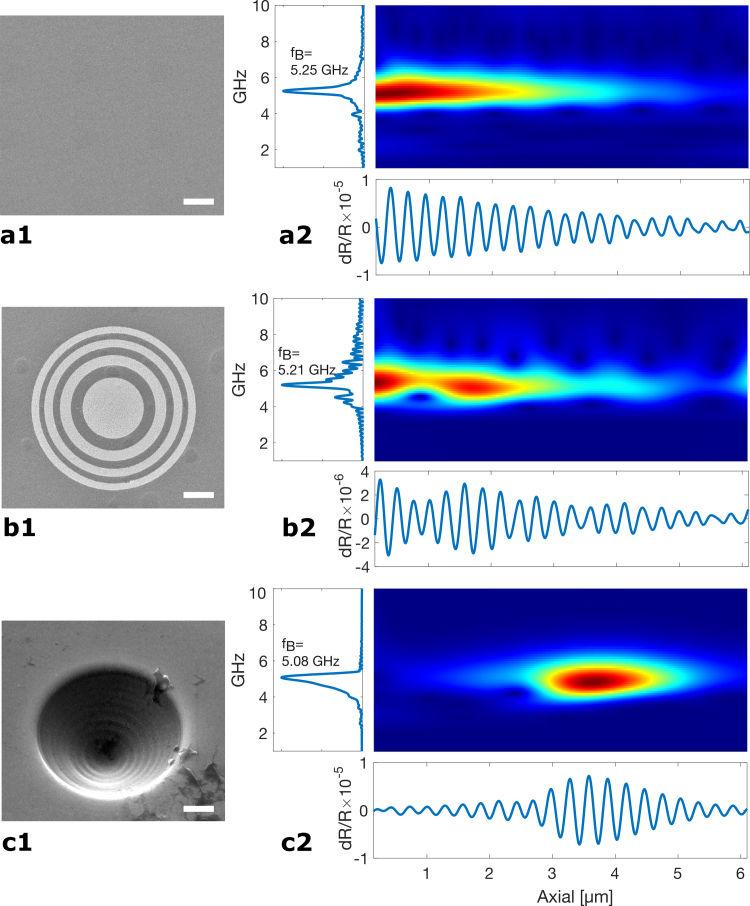
Experimental TRBS traces and frequency analysis of the flat transducer, Fresnel zone plate and concave lens transducer. (a1) Illustration of a conventional flat transducer. (a2) Experimental TRBS trace — modulation depth dR/R (bottom), Fast Fourier Transform (FFT) spectrum indicating the Brillouin frequency ($f_{B}$) peaks (top left) and wavelet visualising the frequency distribution over axial depth (z=vt) (top). (b1) Scanning electron microscope (SEM) image of a Fresnel zone plate lens with a designed focus of 2 μm. (b2) TRBS trace dR/R and frequency analysis of the FZP. (c1) SEM image of a concave lens with a designed focus of 3 μm, corresponding to a travel length equal to the radius of 3.9 μm. (c2) TRBS trace dR/R and frequency analysis of the FZP. Scale bars are 1 μm.

TRBS signals are composed of three elements: (1) a coincidence peak occurring at t=0 caused by a transient change in reflectivity as the pump light is absorbed in the metal film, (2) a slow decaying thermal response and (3) acoustic signals dominated by a strong Brillouin scattering component at around 5 GHz. The coincidence peak is located and cropped from the signal window of interest (and this window is made to begin at t=0), and the slow thermal background is removed by subtracting a low order polynomial fit. The resulting signal is filtered using a bandpass of 3 to 10 GHz, isolating the Brillouin oscillations. The peak frequency of these is identified in the frequency domain using a fast Fourier transform (FFT). Both Brillouin oscillations and the signal in the frequency domain are displayed in Fig. 3 (a2, b2 and c2). To capture the focusing phenomenon along the acoustic time-of-flight – which is equivalent to the axial depth dimension – time–frequency analysis is required to track both the instantaneous frequency and amplitude of the signals. Time–frequency analysis has been widely used for extracting depth-resolved information in TRBS as has been reported previously [32], [33], [41], [42]; here we use the continuous wavelet transform with a complex Morlet wavelet as the mother wavelet.

## 3 Results

By integrating the approach of generating focused coherent acoustic waves using various transducers with TRBS techniques, we demonstrate continuous optical monitoring of focused GHz acoustic fields in water in the following experimental results section. Firstly, we present central TRBS signals from different structures, followed by a discussion of the lateral optical and acoustic PSFs, and finally, line scans to show the lateral performance of the proposed lenses.

### 3.1 TRBS signals

Fig. 3 presents experimental results comparing the performance of a planar transducer (row a), a FZP (row b) and a concave lens (row c). Each row consists of: (a1, b1, c1) electron micrographs of the flat, FZP and concave transducers, respectively; (a2, b2, c2) Brillouin frequency spectrum, wavelet plot and TRBS signal taken at the centre of the transducers for the flat, FZP and concave transducers, respectively. The flat transducer (row a) shows an unfocused acoustic field, indicating the exponential decay of the TRBS signal driven by acoustic attenuation. The signal obtained from FZP, exhibits a non-exponential decay envelope, with a higher energy level around the designed focus (2 μm). However, the modulation depth dR/R is lower compared to the flat transducer, only reaching a maximum of less than $4\times 10^{-6}$. The concave lens offers better focusing than the FZP, with a high modulation depth dR/R envelope around the focus reaching $1\times 10^{-5}$, comparable to the initial TRBS from a flat transducer, as indicated by the strong envelope around R=3.9 μm. The probe focus is maximised over the TRBS trace on the oscilloscope.

Both the FZP and concave lenses lead to TRBS signals having a higher amplitude and a Brillouin frequency shifting towards lower frequencies around the designed focal point (when compared with the plane wave device). The frequency shift effect is caused by the wide range of angles between the probe light and the CAPs (Eq. (1)). In the case of the concave lens, a substantial red-shift is observed. If we assume the lens was uniformly excited, we would expect the minimum Brillouin frequency $f_{B}$ to be ∼4.6 GHz due to the wide angle of incidence between the acoustic waves generated at the extreme diameter of the lens and the probe light. However, the lens was not uniformly excited because of the finite size and distribution of the pump beam. Consequently, the central regions of the lens, with a smaller angle between the probe and CAP, produced a $f_{B}$ of ∼5.08 GHz for the entire trace, with a maximum shift to ∼4.82 GHz observed in the experiments.

### 3.2 The lateral direction red-shift

In order to fully assess the lens’ performance in both the axial and lateral directions and check the red-shift in the lateral direction, line scans along both the x and y axes, as well as area scans, were conducted. These scans can provide information for analysing the performance of the concave lens’s focusing ability, thus offering a continuous measure of the acoustic intensity from the conventional transducer area to the lens area. From these scans, a clear intensity increment is observed experimentally.

**Fig. 4:**
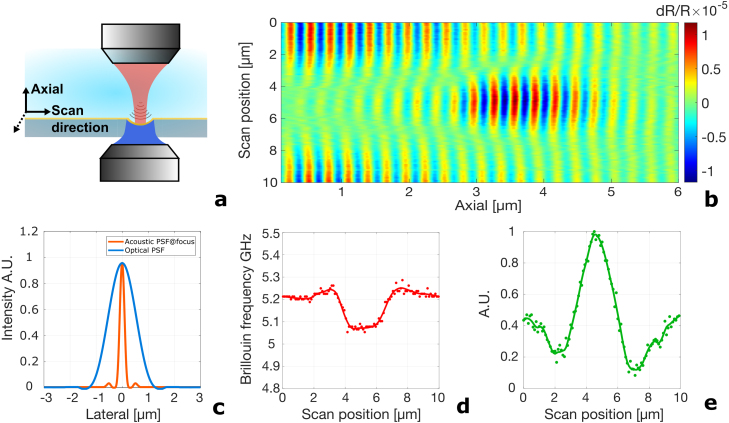
Experimental line scan results across a concave lens. (a) Schematic showing the line scan setup. Pump and probe are focused on each side of the transducer and the TRBS signal is detected in water. The coverslip with concave lenses is sitting in a glass-bottom petri dish on an x–y stage. A 10 μm line across the centre of the lens is scanned with a step of 100 nm. (b) Experimentally measured B-scan plot of the line scan, from 0 to 10 μm of the scan position (y-axis), plotting all the TRBS traces together. The colour bar indicates the modulation depth of the TRBS signal. (c) Theoretical optical probe (blue) PSF and acoustic strain (orange) beam intensity across the focus as a function of the lateral axis. (d) Experimentally measured Brillouin frequency plot across the centre of the lens. (e) The amplitude of the TRBS signal at the focus in green dots, with the same green fitted plot. In both (d) and (e), dots are calculated data points and the lines are fitted traces. A slight increase in the Brillouin frequency can be observed on either side of the focus. This is caused by the superposition of signals from both the flat and concave surfaces near to the edge of the lens.

Fig. 4 presents the line scan results across a concave lens, demonstrating the frequency red-shift and further verifying the focusing. The schematic Fig. 4(a) illustrates the setup where the pump and probe are focused on opposite sides of the transducer, with the TRBS signal detected in water; the coverslip with concave lenses is placed in a glass-bottom petri dish on an x–y stage, and a 10 μm line across the centre of the lens is scanned in 100 nm steps. The B-scan plot Fig. 4(b) shows the TRBS traces over the 0 to 10 μm scan range, with the colour bar indicating the modulation depth of the TRBS signal, revealing strong acoustic focusing by the lens. However, this experimental setup, lacking a sharp acoustic resolution target and with separate movement of the pump and probe, cannot reveal true acoustic focusing, as shown by the orange line in Fig. 4(c). The main limitation is the optical PSF of the detecting objective, represented by the blue line. We observe the result of the convolution of the acoustic beam (orange) and the PSF of the detecting objective (blue) with the current measurement system. The Brillouin frequency plot Fig. 4(d) across the centre, as indicated by the dashed black line in Fig. 4(b), shows a distinct frequency shift at the focal region, caused by the angle of lens generated CAP and the complex reflected probe beam (reflected normal incident probe beam and more complex reflected probe beam due to the existence of concave structure). The amplitude plot of the TRBS shown in Fig. 4(e) illustrates a pronounced peak at the focus, confirming the effective focusing of the concave lens. The focused acoustic beam has a narrow FWHM of around 250 nm as the orange line indicates in Fig. 4(c). The amplitude plot has an FWHM of ∼2.3 μm, which is dominated by the size of the probe beam. These results underscore the lens’ efficacy in concentrating GHz acoustic waves, enhancing the potential for high-resolution acoustic imaging and Brillouin scattering studies.

## 4 Discussion

This study showcases the first continuous optical monitoring of focused GHz acoustic fields in water, achieved by combining the generation of focused coherent acoustic waves across different structures with TRBS techniques. This research investigates a variety of optoacoustic lenses through analysis of both simulation and experimental results. The experimental results show good focusing ability in the performance of a FZP with a design focus of F=2 μm and a high NA of 0.72, as well as in a concave structure with R=3.9 μm and NA of 0.64. The red-shifted Brillouin frequency observed in the experiments also suggests possible new multi-angle TRBS measurement methods.

The demonstrated optoacoustic lenses exhibit significant potential to improve lateral resolution in PLU-based TRBS imaging techniques, particularly for GHz picosecond laser-generated CAPs. However, additional experiments, such as edge detection, are necessary to fully characterise the size of the focused acoustic beam. Furthermore, integrating these lenses into fibre tips could enhance the lateral resolution of fibre-based phonon probe microscopy [29], [33].

While standard concave lenses perform better than FZP, they face more challenges for fabrication at the nano/microscale. Concave acoustic lenses can capture and redirect all the incident pump pulse energy, ensuring minimal loss and maximum energy transfer to the focused acoustic field. The higher energy efficiency, combined with the durability and reusability of these concave structures, enhances their appeal. Fabricated by milling into robust materials like glass substrates, they are designed to withstand repeated use. After experiments, the lenses can be recycled through acid cleaning and re-sputtering of the functional layers, allowing for multiple cycles of reuse without performance degradation. This offers both an economical and practical advantage for extended research, ensuring consistent experimental conditions.

## 5 Conclusions

In conclusion, this study highlights the potential of detecting focused CAPs with Brillouin scattering, by showing the strong focusing of Fresnel zone plates and concave lenses, in both simulation and experiment, used in transducer design within a TRBS system. The findings suggest that focusing using lenses have significant potential to improve lateral resolution in PLU-based TRBS imaging techniques to ∼250 nm. Concave lenses are more efficient compared to FZP and have the practical advantage of being reusable and long-lasting for further research compared with Fresnel zone plate ones. Future work should focus on additional experiments to fully characterise the focused acoustic beam and explore the integration of these concave lenses into a system capable of approaching the lens to the object to be scanned akin to Atomic-force microscopy (AFM). Additionally, improved theoretical models are needed to better understand the complex interactions between the acoustic and optical fields, and to optimise the design and performance of the system in more challenging experimental conditions.

## CRediT authorship contribution statement

**Mengting Yao:** Writing – review & editing, Writing – original draft, Visualization, Validation, Project administration, Methodology, Investigation, Formal analysis, Data curation, Conceptualization. **Rafael Fuentes-Domínguez:** Writing – review & editing, Supervision, Methodology, Investigation, Conceptualization. **Salvatore La Cavera III:** Writing – review & editing, Writing – original draft, Methodology, Investigation, Conceptualization. **Fernando Pérez-Cota:** Writing – review & editing, Writing – original draft, Supervision, Resources, Project administration, Methodology, Investigation, Funding acquisition, Conceptualization. **Richard J. Smith:** Writing – review & editing, Validation, Supervision, Methodology, Investigation, Conceptualization. **Matt Clark:** Writing – review & editing, Validation, Supervision, Resources, Project administration, Methodology, Investigation, Funding acquisition, Conceptualization.

## Declaration of competing interest

The authors declare that they have no known competing financial interests or personal relationships that could have appeared to influence the work reported in this paper.

## Data Availability

Data will be made available on request.
